# The Impact of the Oral and Esophageal Microbiota in EoE and Achalasia

**DOI:** 10.3390/jcm14217502

**Published:** 2025-10-23

**Authors:** Roberta Manente, Gianluca De Caro, Debora Paris, Annabella Tramice, Giovanni Boccia, Pio Zeppa, Fabrizio Chiodo, Paola Iovino

**Affiliations:** 1 San Giovanni di Dio e Ruggi d’Aragona University Hospital, 84081 Salerno, Italy; manente392@gmail.com (R.M.); gianlucadecaro17@gmail.com (G.D.C.); 2 CNR-Institute of Biomolecular Chemistry, Via Campi Flegrei 34, 80078 Pozzuoli, Italy; debora.paris@cnr.it (D.P.); annabella.tramice@cnr.it (A.T.); fabrizio.chiodo@cnr.it (F.C.); 3 Health Hygiene Unit, Department of Medicine, Surgery and Dentistry, University of Salerno, 84081 Baronissi, Italy; gboccia@unisa.it (G.B.); pzeppa@unisa.it (P.Z.); 4 Integrated Care Department of Health Hygiene and Evaluative Medicine, San Giovanni di Dio e Ruggi d’Aragona University Hospital, 84131 Salerno, Italy; 5 Hospital and Epidemiological Hygiene Unit, San Giovanni di Dio e Ruggi d’Aragona University Hospital, 84131 Salerno, Italy; 6 Pathological Anatomy Unit, Department of Medicine, Surgery and Dentistry, Scuola Medica Salernitana, University of Salerno, 84081 Baronissi, Italy; 7 Gastrointestinal Unit, Department of Medicine, Surgery and Dentistry, Scuola Medica Salernitana, University of Salerno, 84081 Baronissi, Italy

**Keywords:** eosinophilic esophagitis, achalasia, microbiota dysbiosis, esophageal microbiota

## Abstract

**Background/Objectives**: Eosinophilic esophagitis (EoE) and achalasia are two chronic esophageal disorders, characterized by inflammatory and neuromotor dysfunction, respectively, that share overlapping immune-inflammatory features. Emerging evidence suggests that dysbiosis of the oral and esophageal microbiota may represent a common determinant in their pathophysiology. This review aims to provide a comparative and integrated overview of microbial and immune alterations in EoE and Achalasia, with potential diagnostic and therapeutic implications. **Methods**: A bibliographic search was conducted on PubMed and Scopus including clinical studies, experimental research, and review articles published between 2015 and 2025. The keywords *Eosinophilic Esophagitis*, *Achalasia*, *Microbiota*, and *Dysbiosis* were used for article selection. **Results**: In EoE, several studies demonstrated increased bacterial diversity with predominance of *Prevotella* and reduction of *Streptococcus*, findings associated with greater inflammatory severity and epithelial barrier dysfunction. Conversely, Achalasia is characterized by reduced microbial diversity and a shift from Gram-positive commensals to Gram-negative taxa capable of activating pro-inflammatory pathways (TLR4-MYD88-NF-κB), leading to neuronal loss and impaired peristalsis. **Conclusions**: Both EoE and Achalasia share the hallmark of dysbiosis, although with distinct immune profiles (Th2 vs. Th17). The identification of specific microbial “signatures” suggests promising perspectives for non-invasive biomarkers and microbiota-targeted therapies, including probiotics and glycan-modulating strategies. Further prospective studies are needed to clarify causal mechanisms and validate microbiota manipulation as a complementary therapeutic approach in esophageal diseases.

## 1. Introduction

The microbiota is the collection of microorganisms that live in close symbiosis with the body, performing essential functions for its homeostasis [1]. The gut microbiota, for example, contributes to the digestion of complex nutrients, produces essential metabolites, such as vitamins and short-chain fatty acids, participates in the maturation of the immune system, and acts as a natural barrier against pathogens [2]. The microbiota’s balance is therefore crucial for health, while its alteration, known as dysbiosis, can promote the development of various diseases [3]. Also in the field of intestinal microbiota, for example, an association has been highlighted with metabolic diseases such as obesity and diabetes, as well as with chronic immune and inflammatory disorders. [4] In recent years, thanks to advanced sequencing techniques, the hypothesis has gained ground that the microbiota may not only be a co-factor in human physiology, but may also play a key role in the etiology of diseases [5]. This is where fecal microbiota transplantation comes in. Already widely used to treat recurrent Clostridioides difficile infections, it is now being studied as a possible therapeutic tool in numerous fields, opening new prospects for personalized medicine [6].

### 1.1. Oral and Esophageal Microbiota

The oral cavity is the gateway to the gastrointestinal tract. Thus, the healthy status of the oral cavity microbiota warrants investigation because it may be associated with gastrointestinal diseases, including eosinophilic esophagitis. *H. pylori* selectively. The oral microbiota represents one of the most diverse microbial systems within the human body. It comprises a wide range of microorganisms, including bacteria, fungi, and viruses, which coexist in a dynamic balance. The approximately 1000 bacterial species of this microbiota are divided into several well-represented and well-known phyla, including Actinobacteria, Firmicutes, and Bacteroidetes. However, there are also less well-represented phyla, such as GN02 (*Candidatus Gracilibacteria*), SR1 (*Candidatus Absconditabacteria*), and TM7 (*Candidatus Saccharibacteria*) [7]. Several studies have demonstrated a correlation between the presence of certain microorganisms and specific diseases of the gastrointestinal tract, while the absence or reduction of beneficial microbial taxa may also predispose to, or influence the course of, such pathologies [8,9]. The translocation of oral bacteria, for instance, into the gastrointestinal tract has been demonstrated to favor gut dysbiosis, promoting the onset of inflammatory bowel diseases such as Crohn’s disease or ulcerative colitis [10]. *Fusobacterium nucleatum*, an oral bacterium, has been identified in colon cancer lesions, suggesting its role in the etiopathogenesis of colorectal cancer [11]. It could be further hypothesized that the evaluation of *Fusobacterium nucleatum* could be used as a biomarker for predicting *Helicobacter pylori* infection in patients with chronic gastritis [8]. Furthermore, it has been demonstrated that *Helicobacter pylori*-positive individuals have a significant increase of *F. nucleatum* in the oral cavity as *H. pylori* selectively adheres and co-aggregates with Fusobacteria. Therefore, it is reasonable to speculate that low levels of *F. nucleatum* can influence (or be influenced by) the absence of *H. pylori*. What is still unclear is whether *H. pylori*-negativity should be considered a consequence of low levels of *F. nucleatum*, or whether the low levels of *F. nucleatum* are due to a lack of *H. pylori* [12]. For a long time, it has been assumed that the esophagus is a sterile environment, largely due to the difficulty of sampling, isolation, and culture. However, in the 1980s, the evidence began to emerge that the esophagus was not sterile [13], and in 1998, a study using gastric aspirate analysis highlighted the close correlation between the oral and esophageal microbiota at the species level [14]. The application of 16S rRNA sequencing has revealed that the microbiota of the esophagus is predominantly comprised of six distinct phyla: Firmicutes, Bacteroidetes, Proteobacteria, Fusobacteria, Actinobacteria, and Saccharibacteria. Of these, the phylum Firmicutes has been identified as the most prevalent [15], while in healthy subjects, the Gram-positive bacteria, including those from the genus *Streptococcus,* predominate. Conversely, Gram-negative bacteria have been observed to prevail in cases of gastroesophageal reflux disease or Barrett’s esophagus [16].

### 1.2. The Pathologies

#### 1.2.1. Eosinophilic Esophagitis

Eosinophilic esophagitis (EoE) is a chronic inflammatory disease characterized by massive activation of the Th2-mediated immune response, the response involved in allergic diseases, resulting in an infiltration of eosinophils into the esophageal mucosa. Landres and colleagues firstly identified this condition in children and subsequently in adults in the late 1970s [17], but only recently has it been classified as a distinct condition in adults. Nowadays, it is officially and globally recognized as a major cause of morbidity in the upper gastrointestinal tract [18]. Diagnostic confirmation of EoE is achieved from multiple esophageal biopsies by detecting ≥ 15 eosinophils per high magnification field (HPF). Frequently, hyperplasia of esophageal squamous epithelium, mostly due to thickening of the basal layer and increased epithelial spongiosis, is also observed. It is hypothesized that these additional histopathological manifestations contribute to esophageal dysfunction and the onset of disease symptoms [19]. Symptoms, which range from mild to severe, can have a major impact on patients’ quality of life. The most reported symptoms are undoubtedly dysphagia and chest or retrosternal pain, while less common symptoms are regurgitation and/or vomiting. In children, symptoms may be less obvious or more challenging to recognize, and they may include difficult swallowing, chest pain, vomiting, and, in more severe cases, even growth retardation. As this symptomatology can also be due to gastroesophageal reflux disease (GERD), it is crucial to distinguish between GERD and EoE. In recent years, a growing number of studies have investigated the causal mechanisms of dysphagia in EoE patients, showing that between 25% and 75% of patients have abnormalities in esophageal motility, suggesting a possible functional and inflammatory role in the genesis of symptoms [20]. The upper gastrointestinal (UGI) endoscopy is an invaluable tool for EoE diagnosis, as it highlights classic features such as concentric rings of the mucosa, longitudinal furrows and narrowing of the esophagus inner diameter. However, a UGI endoscopy alone is insufficient for diagnosis and requires confirmation through biopsies. The latter should reveal increased eosinophilic infiltration in the esophagus (≥15 eos/hpf), while GERD condition may be associated with eosinophilic infiltration, but at a lower density (<10 eos/hpf) [18]. Finally, it should be underlined that a considerable proportion of patients diagnosed with EoE have a medical history marked by atopic diseases, including asthma and allergies. These conditions are characterized by an elevated production of IL-4, IL-5, and IL-13, which are cytokines that play a pivotal role in the Th2-type immune response mechanisms that are typical of atopic diseases. It has been reported that in asthmatic and allergic patients, airway hyperactivity may be associated with altered esophageal activity. Concurrently, multiple sensitizations to food or inhalant allergens are observed in patients diagnosed with EoE [21]. Therefore, EoE should not be regarded as an isolated esophageal disease, but rather as part of a broader spectrum of systemic diseases. Some evidence suggests that eosinophilic infiltration of the esophageal musculature, mediated by T lymphocytes, may contribute to the pathogenesis of achalasia through the release of cytotoxic proteins that damage enteric neurons, suggesting a possible muscular variant of EoE [20]. Numerous cases of patients presenting with both achalasia and EoE have been observed. Furthermore, esophageal motility disorders resembling those of achalasia have been observed to disappear in some patients with EoE who were treated with steroids. Taken together, these findings suggest that achalasia may have an allergic origin at least in some cases. Supporting this hypothesis is a recent retrospective study conducted in the United Kingdom, which highlighted a significant link between achalasia and atopic disorders such as asthma, eczema, and allergic rhinitis [22].

#### 1.2.2. Esophageal Achalasia

Achalasia, a term coined by Hurst in 1927 [23], was first identified by Willis in 1674, who defined it as “blockage of food in the esophagus” [24]. It is an esophageal smooth muscle motility disorder that reduces or eliminates the peristaltic movements of the esophagus and compromises the release of the lower esophageal sphincter (LES) in response to swallowing [25]. This pathology involves a series of symptoms that include mainly dysphagia but also regurgitation, chest pain, and weight loss. Although the causes are not yet well known, the main one appears to be the progressive degeneration of the inhibitory nerve fibers of the myenteric plexus of the esophagus [26]. Genetic factors, viral infections, and autoimmune processes have also been suggested as possible causes contributing to achalasia pathogenesis. Furthermore, recent studies suggest a possible role for esophageal eosinophil infiltration, which, by releasing toxic proteins such as eosinophil cationic protein (ECP), could damage the neurons of the esophageal wall [20]. In addition, esophageal food stasis can attract eosinophils in patients with achalasia. This mimics the mechanism underlying eosinophilic esophagitis, resulting in a risk of misdiagnosis as EoE. Such eosinophil infiltration, in association with the activation of immune cells, such as mast cells, and cytokines, this infiltration can alter the structure of the esophagus, making it more rigid or inflamed [27]. Clinical cases in which EoE patients, showing symptoms similar to achalasia, improve after treatment with steroids are incompatible with the irreversible nature of achalasia, which is caused by the irreversible degeneration of inhibitory neurons in the myenteric plexus of the esophagus. This, therefore, supports the idea that ‘achalasia-like’ forms exist, with an immunological component. Conversely, recent evidence suggests that the degeneration of inhibitory neurons in the myenteric plexus, traditionally considered the primary cause of achalasia, may be a late consequence of chronic allergic inflammation. This implies a substantial change in the definition of the pathology, which would be reclassified from a primary neurodegenerative disease to a potentially reversible immune-mediated condition. Significant histological evidence supports the immunological component underlying the latter hypothesis, namely the marked degranulation of mast cells in the lower esophageal sphincter muscle [22].

### 1.3. Rationale and Aim

This review aims to provide a comparative and integrated analysis of the composition of the microbiota in two esophageal diseases: eosinophilic esophagitis and achalasia. Recent evidence highlights overlapping immuno-inflammatory characteristics and microbial alterations in both conditions, suggesting that they may not only coexist but also represent different manifestations of the same pathophysiological process possibly influenced by the microbiota. In particular, it is being investigated whether dysbiosis may act as a trigger or amplifier of eosinophilic inflammation, neuronal degeneration or esophageal dysfunction. Through a critical synthesis of the literature, the contributions of modern multiomic techniques (metagenomics, metatranscriptomics and metabolomics) and advanced computational tools, which have broadened our understanding of the esophageal microbiome and its interaction with immune pathways, are analyzed. Clarifying these mechanisms could pave the way for new diagnostic strategies and therapies targeting the microbiota in esophageal diseases.

## 2. Materials and Methods

We performed a comprehensive literature search in the PubMed and Scopus databases, using the keywords “eosinophilic esophagitis”, “achalasia”, “microbiota” and “dysbiosis”. Only peer-reviewed articles published in English between 2015 and 2025 were considered. Inclusion criteria included original studies, experimental research, and review articles that directly addressed the role of the oral or esophageal microbiota in esophageal eosinophilia (EoE) or achalasia. Articles reporting data on the microbial composition, associated immunology or inflammatory mechanisms common to the relevant pathologies were also included. Exclusion criteria included case reports, editorials, conference abstracts, and studies not relevant to the esophageal microbiota, as well as works not peer-reviewed or published in languages other than English. After having screened the titles and abstracts of all the articles of interest to us, we finally selected 44 studies, of which 6 specifically focused on the microbiota in EoE and 4 on the microbiota and achalasia. Other works included landmark studies on oral and esophageal microbiota under physiological conditions, research on immune-related mechanisms, and general reviews on the role of dysbiosis in gastrointestinal diseases. Both clinical studies and experimental animal models were considered when they contributed to elucidating microbiota–host interactions in eosinophilic esophagitis or achalasia. The quality and consistency of included studies were evaluated based on the study design, sample size, sequencing platform, and clear definition of disease or experimental model. Disagreements on the relevance of studies selected for inclusion in the review were resolved by the supervisor.

## 3. Interactions Between the Esophageal Microbiota and Esophageal Diseases

### 3.1. Microbiota and EoE

In 2018, Grusell et al. [28] investigated the bacterial diversity in the esophagus of EoE patients and compared it with the bacterial flora of GERD patients and healthy subjects. The study aimed at detecting possible differences to be associated with the pathophysiological processes of the pathologies. Seventeen GERD patients and 10 with EoE underwent endoscopy and biopsies of both the oral cavity and the upper and lower esophagus were collected. The samples were grown on agar media, and the bacterial compositions were compared. The bacterial analysis showed that in both pathologies, the esophagus was poorly colonized, and the most represented bacteria were viridans group streptococci. However, the esophagus in EoE patients showed a greater bacterial diversity, with a median of four bacterial groups or species detected, compared to two in GERD subjects (*p* < 0.0014). No significant differences were observed in the microbiota of the oral mucosa compared to healthy volunteers [28]. Hiremath et al. reported that Prevotella (25%) was the most abundant genus in the salivary microbiota of EoE compared with non-EoE children. However, they observed no statistically significant microbial diversity in the oral cavity microbiota of affected children with EoE compared to controls [29]. It is necessary to clarify, however, that recent taxonomic revisions have separated some species previously assigned to Prevotella, including Prevotella shells, into the new genus Segatella [30] A possible correlation between bacterial composition and severity of the disease was instead the subject of the study by Sasahira et al. [31] Both the oral microbiota and the esophageal microbiota of EoE patients were analyzed. Saliva and brush samples from the distal esophagus were collected and analyzed by 16S sequencing. Also in this case, the Prevotella genus was the most abundant both in saliva and in the esophagus, while a reduction was observed, compared to controls, in the Neisseria genus in saliva and the Streptococcus genus in the esophageal mucosa. Specifically, an interesting inverse correlation was highlighted between the presence of Streptococcus and the severity of the inflammation, measured with the EREFS score: i.e., as the inflammation became more severe, there was a decrease in the quantity of this bacterial genus. Furthermore, the increase in the Prevotella genus, already known for its role in other inflammatory pathologies, could be involved in the pathogenesis of EoE, and this, together with the reduction of Streptococcus, may be associated with the severity of the disease and, therefore, be used as non-invasive biomarkers for diagnosis and monitoring of the pathology [31]. In 2015, evidence was reported on the esophageal microbiota’s role in the pathogenesis and treatment of EoE. Harris et al. [32] compared the microbiota of EoE patients with that of healthy subjects, highlighting a significantly altered microbial composition in patients with active EoE. The collection of esophageal mucosal secretions was carried out using the esophageal string test (EST), the determination of the bacterial load by quantitative PCR, and the analysis of the bacterial communities through the amplification of the 16S rRNA gene and 454 pyrosequencing. A predominance of Haemophilus and Aggregatibacter genera was observed, which are known for their potential pathogenic role, as well as a reduction of bacteria belonging to the genus Firmicutes, typically benevolent commensals. The authors also reported an overall increase in the bacterial load in the esophagus of EoE patients compared to that of healthy subjects. This suggested that eosinophilic inflammation could be linked to a qualitative and quantitative alteration in the bacterial composition. Furthermore, the authors found an association between oral microbiota and esophageal microbiota, hypothesizing that the microbial flora of the mouth could influence the microbial flora of the esophagus [32]. Recent evidence highlighted lipidomic alterations of the esophageal epithelium in patients with EoE, which correlated with IL-5 and IL-13 levels. In particular, an imbalance in the NS/NP ceramide ratio (NP, ceramide with a phytosphingosine chain linked to a nonhydroxy acyl chain; NS, ceramide with a sphingosine linked to a nonhydroxy acyl chain) suggested a possible direct contribution to epithelial barrier dysfunction. Such a dysfunction could favor antigenic penetration and local immune activation, reinforcing the hypothesis that the mucosal microenvironment plays a central role in the pathogenesis of EoE and may be influenced by the local microbial composition [33]. A recent study by Facchin et al. [34] showed how distinctive microbial markers in EoE patients could be identified by using advanced bioinformatics tools. They analyzed saliva, esophageal and gastric samples from EoE patients and healthy controls, identifying 20 sequence variants (ASVs) capable of discriminating between the two groups, with sensitivity and specificity values up to 80%. The MOFA multi-omics approach was able to correlate specific esophageal microbial profiles with factors characterizing the disease. The results were then integrated into the EoE TaMMa web platform, an innovative bioinformatics tool supporting translational research. Their results reinforce the hypothesis that esophageal dysbiosis plays a key role in the pathogenesis of EoE, offering new possibilities for early diagnosis and the development of targeted therapies [34]. Recently, the role of probiotics added to PPIs, corticosteroids, and exclusion diets has been investigated to understand the impact they may have on the composition of the esophageal microbiota. The use of specific probiotic strains could favor a restoration of the microbial composition, helping to reduce inflammation and strengthen the epithelial barrier [35,36]. Although the acquired data are still preliminary, and large randomized studies are lacking, it has been suggested that the modulation of the esophageal microbiome could enhance the efficacy of conventional treatments, reducing side effects and improving adherence to therapy [37]. This approach, which is still at an exploratory stage, opens promising scenarios for the future of integrated EoE management.

### 3.2. Microbiota and Achalasia

Achalasia shares structural and functional abnormalities of the esophagus with EoE, and several studies have investigated the role of the microbiota in its etiopathogenesis. Geng et al. [38] hypothesized that the microbiota in achalasia patients may influence both the development and function of the enteric nervous system. Through the analysis of microbial DNA (16S rRNA sequencing), a reduction in bacterial diversity and a transition from a type I flora (Gram-positive, typical of a healthy esophagus) to a type II flora with predominant Gram-negative bacteria, which are capable of producing lipopolysaccharide (LPS), a stimulator of the innate immune system, was found in patients suffering from achalasia. It is important to note, however, that Gram-negative taxa include not only pathogens but also commensal and mutualistic members of the microbiota. Many of these commensals produce structurally distinct, “modified” forms of LPS [38]. Compared to the canonical, highly inflammatory “endotoxic” LPS, these modified versions exhibit reduced pro-inflammatory activity and are well tolerated by the human immune system [39,40]. In particular, among the main anomalies, a decrease in the Rhodobacter genus-a genus known to express an immunomodulatory LPS that antagonizes TLR4 signaling-and an enrichment of bacteria such as Aquabacterium, Novosphingobium and Lactobacillus was identified in patients with achalasia. To confirm the causal role of these modifications, the development of esophageal dysbiosis was induced in murine models, i.e., mice chronically treated with antibiotics such as ampicillin, in which the presence of a flora similar to that found in patients with achalasia and a reduction in myenteric neurons was found. An increase in lower esophageal sphincter (LES) pressure was observed, as well as the triggering of a local inflammatory response mediated by lamina propria macrophages (LpMs), which activated the TLR4-MYD88-NF-κB pathway with consequent production of pro-inflammatory cytokines (TNF-α, IL-1β and IL-6). The central role of this pathway was confirmed by dysbiosis induced in TLR4 knock-out (TLR4KO) mice, in which no esophageal dysfunction or neuronal loss was found [38]. More recently, both the characterization of the esophageal microbiota and its evolution after treatment have been investigated. In a prospective cohort of 29 patients undergoing endoscopic peroral myotomy (POEM), the esophageal microbial composition was analyzed before and after surgery. The microbial community was dominated by the phyla Firmicutes, Bacteroidetes, Proteobacteria, Actinobacteria, and Fusobacteria, with Streptococcus as the most abundant genus. Despite the significant improvement in nutritional intake after POEM, the esophageal microbial composition did not show substantial changes in the short term, suggesting that dysbiosis may persist even after restoration of peristalsis [41]. Another study compared the oral and esophageal microbiota in achalasia patients, showing significant differences between the two sites. After POEM, although no global changes in microbial structure emerged, a relative increase in Haemophilus and Neisseria was observed, associated with an endoscopic improvement in mucosal inflammation. These observations indicated that, in addition to chronic food stasis, post-treatment ecological niche modification could selectively modulate certain bacterial populations [42]. Recent evidence has linked altered microbiota with impaired systemic lupus erythematosus (SLE) contractile function and the Th17 immune response. In patients with type-II achalasia, hypophosphorylation of myosin light chains (LC20) was found to be related with downregulation of the myosin phosphatase inhibitor protein CPI-17. At the same time, esophageal levels of Th17 cytokines (IL-17A, IL-17F, IL-22, IL-23A) were significantly increased. In details, Actinomyces abundance was positively correlated with IL-23A levels, while Dialister correlated with IL-17A, IL-17F and IL-22, suggesting a possible direct role of certain bacterial species in the activation of pro-fibrotic inflammatory pathways and neuromuscular dysfunction. Furthermore, the inoculation of the esophageal intraluminal environment (conditioned medium) in mice increased IL-17F, strengthening the hypothesis of a causal effect of the microbiota on the inflammatory phenotype [43].

## 4. Conclusions and Future Directions

**Figure 1:**
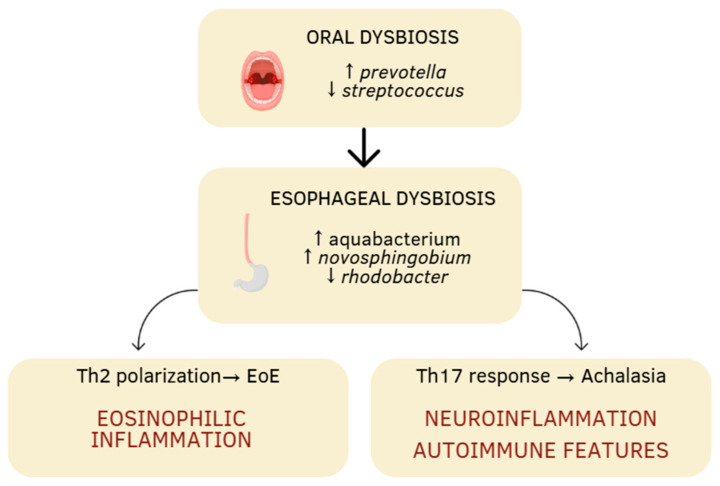
Schematic representation of the oral–esophageal microbiota–immune axis continuum. Oral dysbiosis characterized by an increase in *Prevotella* and a decrease in *Streptococcus* may extend to the esophagus, where further microbial shifts (*Aquabacterium*, *Novosphingobium*, *Rhodobacter*) contribute to local immune activation. These alterations can promote distinct immunological pathways: Th2 polarization leading to eosinophilic inflammation in eosinophilic esophagitis (EoE), and Th17-driven neuroinflammation and autoimmune features in achalasia. The arrows indicate an increase or decrease.

**Figure 2:**
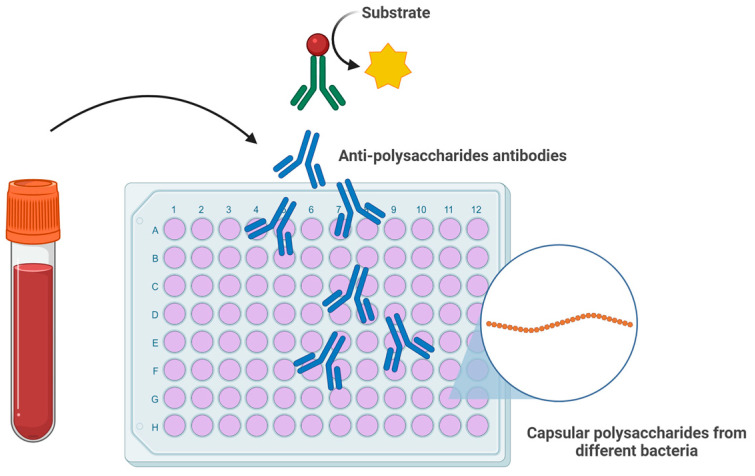
Schematic representation of an ELISA-based assay for the detection of anti-polysaccharide (anti-glycan) antibodies. Serum antibodies bind to immobilized bacterial capsular polysaccharides, followed by detection with enzyme-conjugated secondary antibodies and substrate conversion. Such assays could be adapted to profile anti-glycan antibody responses in patients with EoE or achalasia, potentially serving as biomarkers for disease stratification, monitoring, and prediction of treatment outcomes.

EoE and achalasia, despite their clinical and immunological differences, could share some common underlying mechanisms influenced by the microbiota. The comparative analysis of the microbiota in these two disease states suggests that dysbiosis is a shared feature, although with different underlying mechanisms and manifestations. For EoE, literature data indicate that changes in the microbiota involve increased bacterial diversity, especially with a dominance of the *Prevotella* genus and a decrease in *Streptococcus.* Additionally, the rise in potentially pathogenic bacteria like *Haemophilus* and *Aggregatibacter*, along with an overall increase in bacterial load, indicates significant qualitative and quantitative alterations. With regard to achalasia, several studies have suggested a reduction in microbial diversity and a possible shift from a Gram-positive commensal flora towards a Gram-negative flora, which may contribute to innate immune activation, partly through lipopolysaccharide (LPS)-mediated pathways. This dysbiosis is linked to chronic inflammation, activation of the TLR4-MYD88-NF-κB pathways, neuronal loss, and impaired peristalsis. Furthermore, the association between specific bacterial strains and the activation of the pro-fibrotic Th17 response supports the idea that the microbiota may directly contribute to disease progression. It is therefore reasonable to hypothesize the existence of specific microbial ‘signatures’ and microbial “shifts” in EoE and achalasia, with potential diagnostic and predictive implications. These differences are summarized in Table 1, which compares the main microbiological, immunological and pathophysiological aspects of EoE and achalasia. A schematic representation of the oral–esophageal microbiota–immune axis continuum is illustrated in Figure 1. Such signatures may prove particularly valuable in cases of esophageal motility disorders resembling achalasia in certain patients with EoE, as well as in conditions characterized by eosinophilic infiltration and immune cell activation, which may likewise be observed in achalasia. Emerging evidence suggests that anti-glycan antibodies may serve as diagnostic and prognostic markers in immune-mediated disorders. Glycans play a crucial role in host-microbiota host-pathogens interactions, and their dynamics and/or dysregulation have been linked to autoimmune and inflammatory conditions, highlighting the potential of anti-glycan antibodies in distinguishing disease phenotypes, particularly in gastrointestinal disorders [44]. Prospective hypothesis is that in Th2-mediated diseases such as EoE, microbial dysbiosis may trigger aberrant immune responses, including the production of anti-glycan antibodies, which could correlate with disease severity or treatment response. Similarly, in achalasia, where autoimmune mechanisms have been implicated, anti-glycan profiling could help identifying subsets of patients with distinct pathophysiological pathways. The clinical importance of antibodies as biomarkers is exemplified by existing assays like the Platelia Aspergillus IgG (Bio-Rad), which detects fungal-specific antibodies to diagnose chronic pulmonary aspergillosis. By analogy, anti-glycan antibodies panels could be developed to stratify EoE and achalasia patients, predicting therapeutic outcomes or monitoring disease progression (Figure 2). Collectively, the interplay between microbial dysbiosis, anti-glycan antibodies, and immunomodulatory LPS from microbiota opens up different novel opportunities for clinical translation. Firstly, this knowledge paves the way for microbiota-targeted interventions. These could range from pre/probiotic formulations designed to restore a homeostatic microbial community to more precise approaches, such as leveraging specific LPS structures or glycan motifs as immune modulators to actively suppress pathogenic inflammation in EoE or interrupt autoimmune triggering in achalasia. Secondly, the profiling of anti-glycan antibodies presents a clear path toward novel diagnostic and prognostic tools. The development of a serological panel could improve patient management. Such a test would enable the stratification of patients into distinct sub-phenotypes based on their underlying immune triggers and predict responsiveness to therapies. LPS, a key pathogen-associated molecular pattern (PAMP) of Gram-negative bacteria, exhibits remarkable structural heterogeneity that dictates its immunostimulatory or immunosuppressive responses. The lipid A domain, core oligosaccharide and O-antigen chains collectively determine whether LPS triggers robust pro-inflammatory responses (via TLR4/MD-2 signaling) or induces immune tolerance or immune modulation [39]. The different LPS glycan structures can dramatically alter host immune recognition. In EoE, dysbiosis-driven exposure to specific LPS variants (like an hypoacylated lipid A) may promote Th2-skewed inflammation, exacerbating the eosinophil recruitment. In achalasia, where microbial triggers of autoimmunity are suspected, certain LPS structures could foster molecular mimicry and pathogenic autoantibody production.

While the observed taxonomic shifts in the oral and esophageal microbiota in EoE and achalasia are convincing, the current evidence possesses significant limitations that preclude definitive conclusions on the link between causality and mechanisms. A primary limitation is the correlative nature of most of the reported studies, which identifies microbial associations but falls short of demonstrating a direct pathogenic role. Furthermore, the reliance on stool samples for some inferences introduces potential confounding, as the gut microbiota may not accurately reflect the disease-relevant micro-environment at the site of pathology. Crucially, the field is still developing in elucidating the underlying molecular mechanisms. The functional impact of specific bacterial losses or gains on disease initiation, severity, and progression remains largely speculative and requires further investigation.

Although this review integrates both clinical and experimental evidence to provide a comprehensive overview, it is important to acknowledge that findings from murine models may not be fully translatable to human disease. Additionally, the possibility of publication bias cannot be excluded, and factors such as antibiotic exposure, dietary habits, and environmental influences may act as confounders affecting microbiota composition across studies.

Overall current literature suggests that the esophageal microbiota may significantly influence the onset and progression of both diseases. The possibility of modulating the microbiota, for example through targeted probiotics, represents innovative and integrated therapeutic options. However, further prospective and randomized studies are needed to better understand the causal role of these microbiota alterations and to develop microbiota manipulation strategies as a complementary treatment for esophageal diseases.

While the emerging associations between specific microbial taxa, immune pathways (Th2/Th17), and disease persistence are compelling, causal relationships remain hypothetical, as most available studies are observational. Future longitudinal and interventional investigations with standardized sampling and controlled probiotic approaches are required to confirm these causal links and assess therapeutic potential.

Furthermore, advances in glycoengineering could enable the manipulation of LPS structures, offering new potential therapeutic strategies such as engineered probiotics or synthetic glycans to modulate TLR4 signaling and restore immune balance in these esophageal disorders. Preliminary supporting data are already being collected.

## Figures and Tables

**Table 1:** Microbiota–pathology relationship: comparison between eosinophilic esophagitis and achalasia in terms of microbial diversity, immune response, pathophysiological consequences, persistence of dysbiosis, and therapeutic perspectives. Data summarized from references [28,29,30,31,32,33,34,35,36,37,38,39,40,41,42,43].

Aspect	Eosinophilic Esophagitis	Achalasia
**Microbial diversity**	Increased	Reduced
**Altered dominant flora**	* **↑ prevotella,** * * **↑ haemophilus,** * * **↑ aggregatibacter,** * * **↓ streptococcus** *	* **↑ aquabacterium,** * * **↑ novosphingobium,** * * **↑ lactobacillus,** * * **↓ rhodobacter** *
**Type of immune response**	prevalence Th2 (↑ IL-4, IL-5, IL-13)	prevalence Th17 (↑ IL-17A, IL-17F, IL-22, IL-23A) Activation TLR4-MYD88-NF-κB
**Physiopathological consequences**	epithelial barrier dysfunction, eosinophilic inflammation	neuronal loss of the myenteric plexus, esophageal motor dysfunction
**Persistence of dysbiosis after treatment**	not clearly documented (but possible)	persistent even after POEM ^1^
**Therapeutic prospects**	probiotics in experimental phase to restore the barrier and modulate inflammation	probiotics or targeted interventions to limit immune activation

^1^ POEM, abbreviation for Peroral endoscopic myotomy. Color coding: blue = Gram-positive; red = Gram-negative bacterial taxa. The arrows indicate an increase or decrease.

## Data Availability

No new data were created or analyzed in this study. Data sharing is not applicable to this article.
